# Muscle Fatigue Enhance Beta Band EMG-EMG Coupling of Antagonistic Muscles in Patients With Post-stroke Spasticity

**DOI:** 10.3389/fbioe.2020.01007

**Published:** 2020-08-18

**Authors:** Le-Jun Wang, Xiao-Ming Yu, Qi-Neng Shao, Ce Wang, Hua Yang, Shang-Jun Huang, Wen-Xin Niu

**Affiliations:** ^1^Physical Education Department, Sport and Health Research Center, Tongji University, Shanghai, China; ^2^Key Laboratory of Spine and Spinal Cord Injury Repair and Regeneration of Ministry of Education, Orthopaedic Department, Tongji Hospital, Tongji University School of Medicine, Shanghai, China; ^3^Department of Rehabilitation, Shanghai Seventh People’s Hospital, Shanghai University of Traditional Chinese Medicine, Shanghai, China

**Keywords:** antagonist muscle, coactivation, EMG, muscle fatigue, post-stroke spasticity

## Abstract

There is a significant influence of muscle fatigue on the coupling of antagonistic muscles while patients with post-stroke spasticity are characterized by abnormal antagonistic muscle coactivation activities. This study was designed to verify whether the coupling of antagonistic muscles in patients with post-stroke spasticity is influenced by muscle fatigue. Ten patients with chronic hemipare and spasticity and 12 healthy adults were recruited to participate in this study. Each participant performed a fatiguing isometric elbow flexion of the paretic side or right limb at 30% maximal voluntary contraction (MVC) level until exhaustion while surface electromyographic (sEMG) signals were collected from the biceps brachii (BB) and triceps brachii (TB) muscles during the sustained contraction. sEMG signals were divided into the first (minimal fatigue) and second halves (severe fatigue) of the contraction. The power and coherence between the sEMG signals of the BB and TB in the alpha (8–12 Hz), beta (15–35 Hz), and gamma (35–60 Hz) frequency bands associated with minimal fatigue and severe fatigue were calculated. The coactivation ratio of the antagonistic TB muscle was also determined during the sustained fatiguing contraction. The results demonstrated that there was a significant decrease in maximal torque during the post-fatigue contraction compared to that during the pre-fatigue contraction in both stroke and healthy group. In the stroke group, EMG-EMG coherence between the BB and TB in the alpha and beta frequency bands was significantly increased in severe fatigue compared to minimal fatigue, while coactivation of antagonistic muscle increased progressively during the sustained fatiguing contraction. In the healthy group, coactivation of the antagonistic muscle showed no significant changes during the fatiguing contraction and no significant coherence was found in the alpha, beta and gamma frequency bands between the first and second halves of the contraction. Therefore, the muscle fatigue significantly increases the coupling of antagonistic muscles in patients with post-stroke spasticity, which may be related to the increased common corticospinal drive from motor cortex to the antagonistic muscles. The increase in antagonistic muscle coupling induced by muscle fatigue may provide suggestions for the design of training program for patients with post-stroke spasticity.

## Introduction

The ability of the central nervous system to appropriately control agonistic and antagonistic muscles is a key mechanism to maintain body coordination during human voluntary movements and postural adjustments, which can be damaged by post-stroke spasticity (Tamburella et al., 2017). Spasticity is a common complication of stroke and affects up to 40% of patients with hemiplegia (Wissel et al., 2013). It results from the decreased inhibition or facilitation of hypertonia after stroke as a result of motor impairment (Wang et al., 2018). The decreased inhibitory input to the motor unit leads to uncoordinated activities of antagonist muscles during volitional movement (Turpin et al., 2017; Levin et al., 2018).

Neuromuscular fatigue has the tendency to arise when performing physical activities after stroke, which has received little attention in clinical rehabilitation research (Boudarham et al., 2014). It can be defined as a reversible reduction in the neuromuscular system’s capacity to generate force or power (Vollestad, 1997), which encompasses a number of changes occurring at both the central and peripheral levels (Enoka et al., 2011; Wang et al., 2017). The relationship between agonist and antagonist muscles during muscle fatigue has been focused on, and significant muscle fatigue-induced interaction changes have been reported previously (Duchateau and Baudry, 2014; Arellano et al., 2016). Particularly, previous studies have revealed an increased interconnection between synchronized cortical neurons and the motoneuron pool of antagonist muscles as a result of fatigue (Wang et al., 2015). However, whether neuromuscular fatigue influences the control of antagonist muscles in patients with post-stroke spasticity remains unclear.

Frequency-based electromyography (EMG) assessment has been used as an effective tool to explore the motor control strategy of movement. In particular, EMG–EMG coherence analysis is a method of reflecting the similarity of two signals in frequency domain and has been applied to evaluate the common synaptic input to co-contracting muscles or to two parts of the same muscle from branches of last order neurons to spinal motoneurons (De Luca and Mambrito, 1987; Keen et al., 2012). Previous researches have suggested that inter- and intramuscular coherence in beta (15–35 Hz) and gamma (35–60 Hz) bands are predominantly driven by the motor cortex (Baker et al., 1997), whereas coherence in the alpha (8–12 Hz) band is influenced by multifactors (McKiernan et al., 2000). In previous research, EMG-EMG coherence analysis has been widely used to reflect neuromuscular control mechanisms of movement and to gauge the functional integrity of corticomotor tracts while patients with stroke complete motor tasks (Lodha et al., 2017; Wang et al., 2019).

Based on these studies, we wondered whether the coupling and controlling strategies of antagonistic muscles in patients with post-stroke spasticity can be influenced by muscle fatigue. The current study was designed to verify this hypothesis. EMG activities of the biceps brachii (BB) and triceps brachii (TB) were recorded from both patients with post-stroke spasticity and healthy subjects during fatiguing isometric elbow flexion contraction. The amount of antagonist activation and EMG-EMG coherence between the BB and TB were compared between the first (stage 1 with minimal fatigue) and second halves (stage 2 with severe fatigue) of the contraction.

## Materials and Methods

### Participants

Twelve healthy adults (12 men; age: 21.1 ± 3.4 years; height: 177.6 ± 7.7 cm; weight: 66.25 ± 9.5 kg) and 10 patients with chronic hemiparesis (7 men and 3 women; age: 60.5 ± 9.1 years; height: 169.9 ± 8.8 cm; weight: 69.9 ± 11.3 kg) participated in this study. All healthy subjects reported no known neuromusculoskeletal impairments and were right-handed. The inclusion criteria for the patients with stroke were as follows: (1) hemiplegia secondary to an ischemic or hemorrhagic stroke, (2) at least 6 months post-stroke, (3) residual voluntary elbow flexion force, (4) spastic hypertonia in elbow flexors of the impaired side, rated as Modified Ashworth Scale (MAS) less than 3, and (5) able to understand and follow instructions related to the experiment. All subjects were fully informed of all procedures and risks associated with the experimental tests before providing their informed written consent to participate. The experiment was approved by the Ethics Committee of Tongji University.

### Experimental Tasks

The experiment consisted of three motor tasks. First, participants performed isometric maximal elbow flexion contraction test for three times with 5 min rest after each test, in order to acquire the maximal isometric elbow flexion torque of each subject without muscle fatigue. Second, after 5-min rest, each participant performed a sustained elbow flexion fatiguing contraction at 30% maximal elbow flexion force for as long time as possible. Lastly, as soon as the fatiguing elbow flexion contraction task was complete, the subjects were instructed to perform another isometric maximal elbow flexion contraction test. All motor tasks were finished with the affected limb.

**FIGURE 1:**
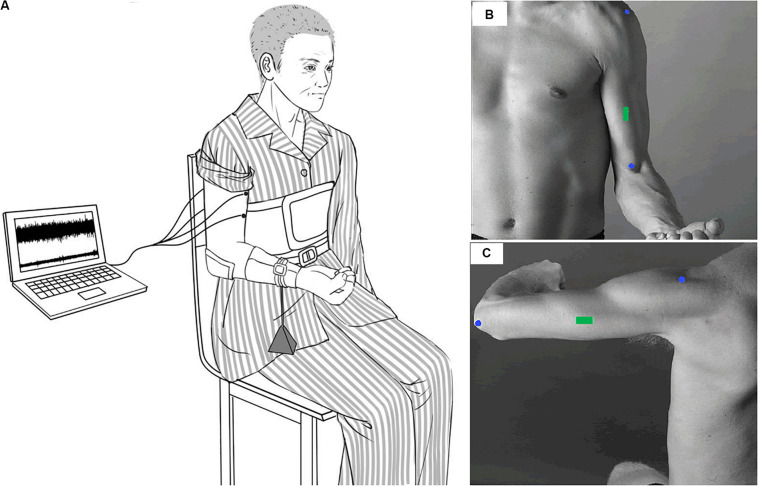
Experimental setup. Participants sat with the upper arm vertically placed and the elbow angle keeping in 90°. The right forearm was positioned parallel to the ground and supinated. During the sustained elbow flexion contraction, a weight was suspended from the distal part of the right forearm so as to produce a target force of 30% maximal elbow flexion force **(A)**. Surface EMG signals were recorded from the BB and TB of the affected side. The EMG sensors of the BB and TB were placed on the green rectangle of **(B,C)** according to SENIAM recommendations.

In the experiment, the participants sat with the upper arm vertically placed, and the elbow angle was maintained at 90°. The right forearm was positioned parallel to the ground and supinated. The participants were instructed to maintain their posture by flexing the elbow with the elbow joint maintained as close as possible to 90° until they experienced exhaustion and were no longer able to continue the contraction. The participants were verbally vigorously encouraged to continue the sustained contraction for as long as possible. During the sustained contraction, surface EMG signals of the BB and TB muscles of the affected limb were recorded. The experimental setup has been depicted in Figure 1.

### Data Collection

The elbow flexion torque was measured using a torque sensor (TRS-500, Transducer Techniques, Temecula, CA). The sensor was located in line with the center of the rotation of the active elbow joint. Surface electromyographic signals were recorded by bipolar surface electrodes of NeuroScan system (NeuroScan Inc., El Paso, TX). Two pairs of electrodes were placed over the belly of the right BB and TB muscles with a center-to-center inter-electrode distance of 2 cm. A common reference electrode was placed to the left of the processus mastoideus. The skin was shaved and cleaned with alcohol wipes before filling the electrodes with conducting gel and fixing. Medical adhesive tape and plastic casts were applied to fix the electrodes. The EMG signals were amplified, band-pass filtered (3–1000 Hz), digitized (2000 samples/s), and acquired by using the NeuroScan system.

### Data Analysis

Data were analyzed off-line by custom-written programs using MATLAB R2016a software (Mathworks Inc., Natick, MA).

#### Data Preprocessing

The raw EMG signals recorded from both the BB and TB muscles were band-pass filtered offline at 5–500 Hz using a fourth order zero-phase-shift Butterworth filter. Then, the filtered surface EMG (sEMG) signals of both the BB and TB muscles were further full-wave rectified using the Hilbert transform according to the research of Farina et al. (2013). Filtered and rectified EMG signals were adopted in the latter coherence analysis process, while filtered-only EMG were used when calculating EMG median frequency, power and coactivation ratio.

#### EMG Median Frequency, Power, and Coactivation Ratio

The EMG median frequency and power spectrum based on Fourier transform of filtered-only EMG signals were calculated for non-overlapping 2.048-s epochs during the sustained fatiguing contraction and were averaged for each epoch during the first and second halves of the sustained elbow flexion contraction. The EMG power in the alpha (8–12 Hz), beta (15–35 Hz), and gamma (35–60 Hz) frequency bands were acquired during the first and second halves of the sustained elbow flexion contraction.

The coactivation ratio of the antagonistic TB muscle, described as the quotient of the TB EMG amplitude divided by the sum of the BB and TB EMG amplitudes, was also calculated for non-overlapping 2.048-s epochs during the sustained fatiguing contraction and time normalized to 20 points for each subject and then averaged for all subjects.

#### EMG-EMG Coherence

The filtered and rectified sEMG signals of each participant were equally divided into two segments: the first and second halves of the contraction. Coherence analysis was conducted on each half segment between EMG signals recorded from the BB and TB muscles. The coherence was calculated for a segment length of 2048 samples with 50% overlap, using a Hanning window, as suggested by previous research.

The magnitude squared coherence, Cxy(f) between the two EMG signals (rectified EMG signals) recorded from the BB and TB muscles, x(t) and y(t), for a given frequency f was calculated as follows:

(1) $$C_{x\,y}\,\left(f\right)=\frac{{\left|S_{x\,y}\,\left(f\right)\right|}^{2}}{S_{x\,x}\,\left(f\right).S_{y\,y}\,\left(f\right)}$$

where Sxy(f) is the cross spectrum and Sxx(f) and Syy(f) are the auto spectra of x(t) and y(t), respectively.

The significance level of the coherence spectrum was calculated based on the methods described by Terry and Griffin (2008). The coherence areas in the alpha, beta and gamma bands greater than the upper 95% confidence interval of the coherence spectrum were computed.

### Statistical Analysis

Statistical analysis was performed using IBM SPSS 20.0 (SPSS Inc., Chicago, IL). Normality was tested using the Kolmogorov–Smirnov test. A repeated-measures general linear model was used to determine the difference between the maximal torque and median frequency. Pearson’s cross-correlation analysis was employed to determine the relationship between the coactivation ratio of antagonistic muscles and the contraction duration time. The non-parametric Wilcoxon test was adopted to test the difference in EMG power and coherence between the first and second halves of the contraction. All significance thresholds were set at α = 0.05.

## Results

### Maximal Torque, Median Frequency, and Antagonist Muscle Coactivation

**FIGURE 2:**
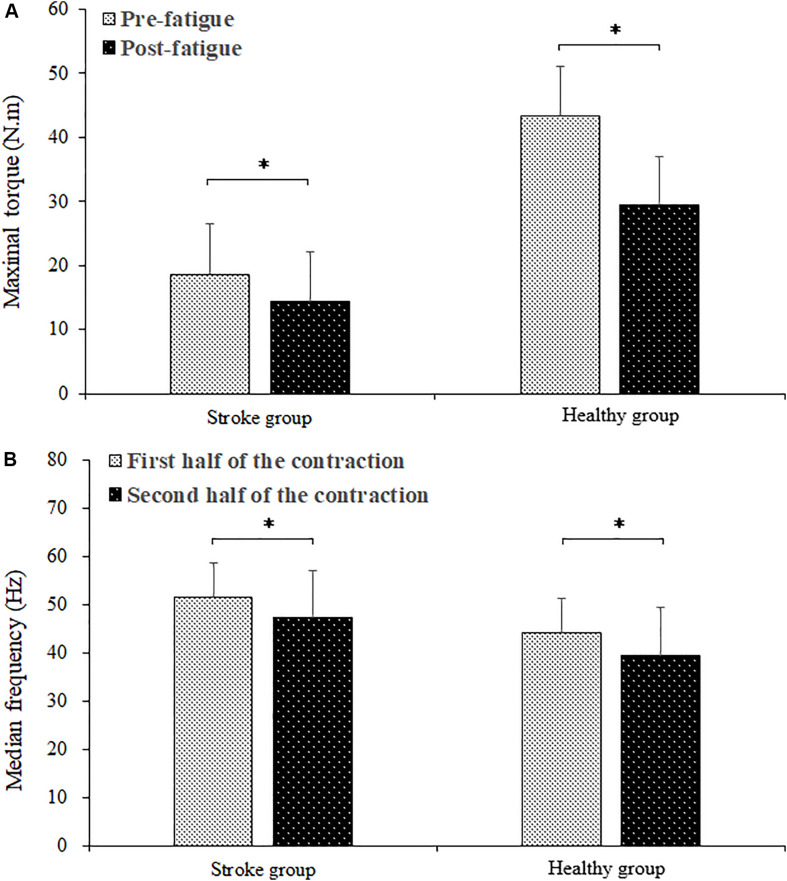
Comparison of maximal torque between the pre- and post-fatigue **(A)** and the BB EMG median frequency between the first and second halves **(B)** of the fatiguing contraction. ^∗^ demonstrated a significant difference of observed index between the pre- and post-fatigue conditions.

The fatiguing contraction times of the stroke group and healthy group subjects were averages of 220.66 ± 109.19 s and 258.58 ± 51.13 s, respectively. There was no significant difference between the fatiguing contraction times of the stroke and healthy group subjects (*P* = 0.296). Figure 2 shows the comparison of maximal torque between pre- and post-fatigue and EMG median frequency of the BB muscle between the first and second halves of the fatiguing contraction of the stroke and healthy group subjects. The maximal torques of the healthy group subjects were significantly higher than those of the stroke group subjects (*F* = 30.758, *P* = 0.000). A significant group by fatigue interaction was found (*F* = 35.771, *P* = 0.000). For both the stroke and healthy group subjects, there was a significant decrease in the maximal torque during post-fatigue contractions compared to that during pre-fatigue contractions (stroke group: *P* = 0.000; healthy group: *P* = 0.000). The median frequency decreased significantly during the second half of the fatiguing contraction compared to that during the first half contraction (*F* = 46.332, *P* = 0.000). A higher median frequency was found in the stroke group than in the healthy group subjects (*F* = 8.050, *P* = 0.010). No significant group by fatigue interaction was present (*F* = 0.096, *P* = 0.760).

**FIGURE 3:**
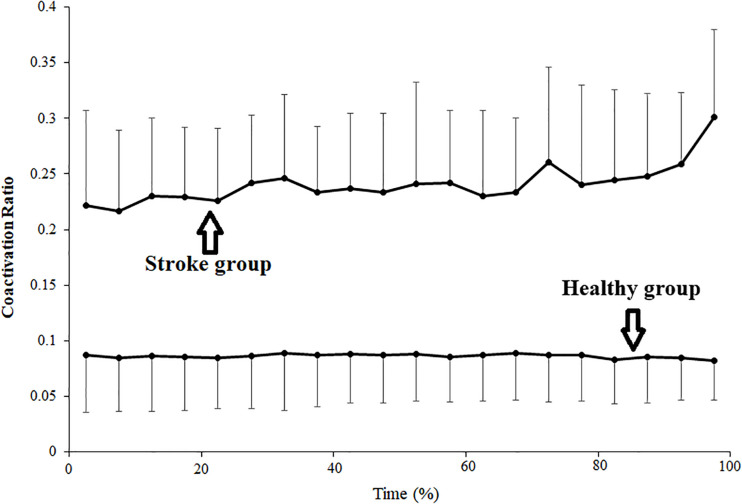
Changes of average coactivation ratio plotted as a percentage of contraction time during the sustained fatiguing contraction. The coactivation ratio was calculated as quotient of the antagonistic muscle TB EMG amplitude divided by the sum of agonistic muscle BB EMG amplitude and TB EMG amplitude.

Changes in the average coactivation ratio among stroke and healthy subjects for each 5% duration times are shown in Figure 3. In the stroke group, the coactivation ratio was 0.222 ± 0.085 in the first 5% of the contraction duration and increased progressively during the sustained fatiguing contraction, reaching 0.301 ± 0.079 in the last 5% contraction duration. In the healthy group, the coactivation ratios were 0.087 ± 0.052 in the first 5% contraction duration and 0.082 ± 0.036 in the last 5% of contraction duration. Pearson’s correlation analysis was used to observe the correlation between coactivation ratio and contraction duration, and a significant increasing tendency of the contraction ratio with the change in contraction duration was found in the stroke group (*r* = 0.183, *P* = 0.010), while no significant correlation was found between the contraction ratio and contraction duration in the healthy group (*r* = −0.011, *P* = 0.868).

### EMG Power of Agonist and Antagonist Muscles

**FIGURE 4:**
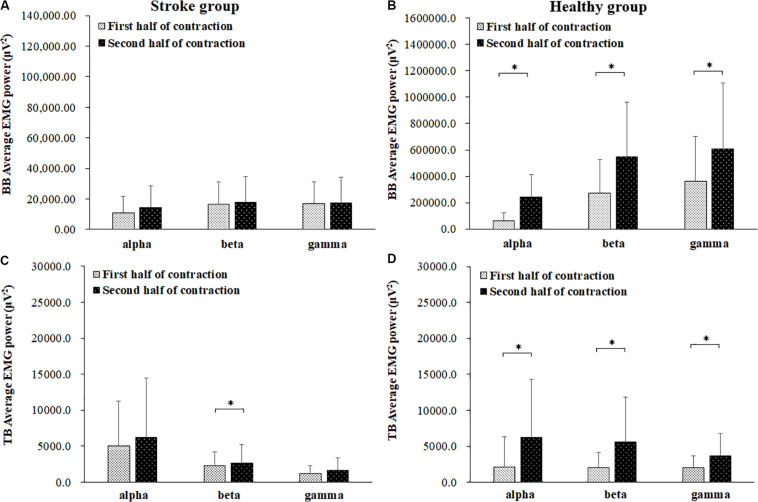
Comparisons of the average EMG power of BB **(A,B)** and TB **(C,D)** muscles of the stroke **(A,C)** and healthy **(B,D)** groups. in the alpha (8–12 Hz), beta (15–35 Hz), and gamma (35–60 Hz) frequency bands between the first (light bar) and second (dark bar) halves of the fatiguing contraction. A significant increase of EMG power was found at BB muscle in the alpha frequency band and TB muscle in beta frequency band. ^∗^ demonstrated a significant difference of observed index between the first and second half of contraction.

The average EMG power of the BB and TB muscles in the alpha, beta and gamma frequency bands during the first and second halves of the contraction in the stroke and healthy group were presented in Figure 4. In the stroke group, the power of the TB muscle in the beta frequency band was significantly higher during the second half of the contraction than during the first half of contraction (*P* = 0.046). In the healthy group, the powers of both the BB and TB muscles in the alpha, beta and gamma frequency bands showed significant increases during the second half of the contraction compared to those during the first half of contraction (For BB, alpha: *P* = 0.000, beta: *P* = 0.000, gamma: *P* = 0.006; For TB: alpha: *P* = 0.016, beta: *P* = 0.016, gamma: *P* = 0.010).

### Coherence Analysis

**FIGURE 5:**
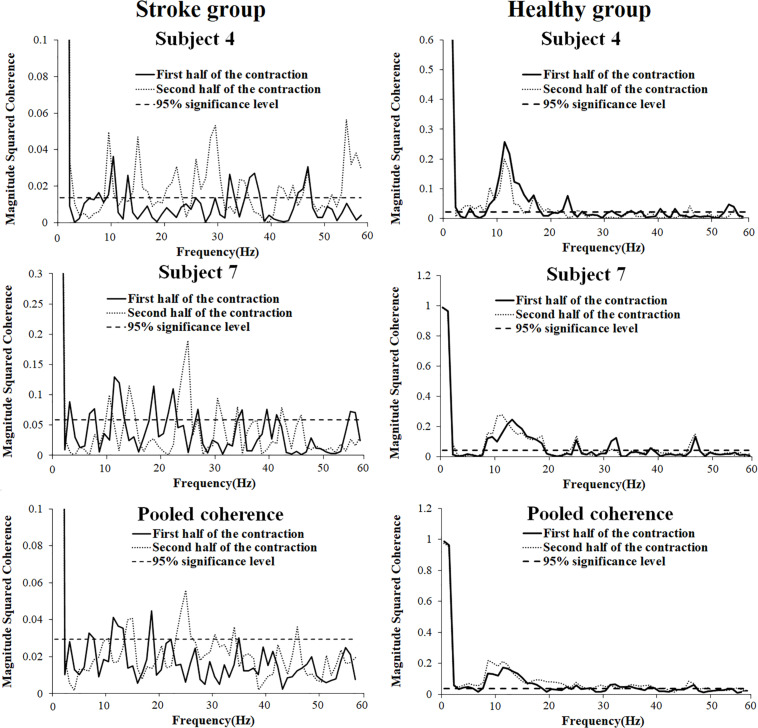
Representative (subject 4, 7, and 10) and pooled coherence spectra of the BB and TB sEMG signals during the first (thick line) and second (thin line) halves of the fatiguing contraction.

In order to present the characteristics of the coherence spectra and provide clues to verify the impact level of cross-talk on the results, representative coherence spectra of two subjects as well as pooled coherence spectra of all the subjects in the stroke and healthy group were presented in Figure 5. It can be seen from the figure that in the stroke group, significant intermuscular coherence was found in distinct frequency bands rather than in a broadband spectrum. Significant coherence was mainly appeared in beta frequency band in the pooled coherence spectrum and was higher in the second half of contraction compared to the first half contraction. In the healthy group, significant coherence was observed across a broad range of frequencies and prominent peak coherence values were found around 10 Hz during the first and second halves of contraction. The coherence spectra showed had very similar values between the first and second halves of the contraction at the same frequency point in the healthy group.

**FIGURE 6:**
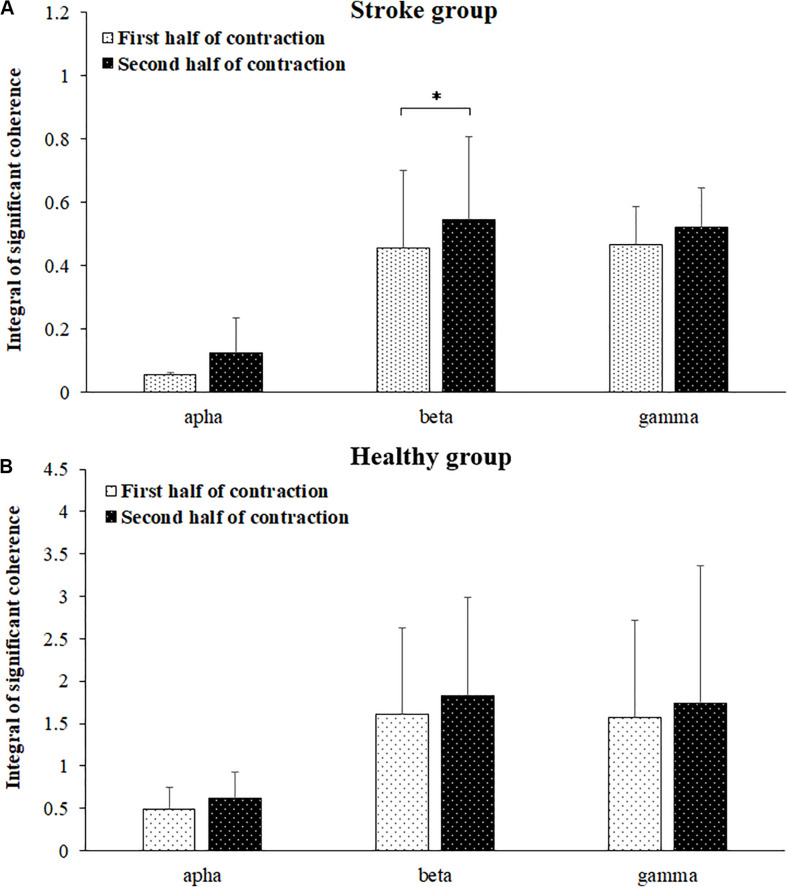
Comparisons of significant coherence integral for the stroke **(A)** and healthy **(B)** groups in the alpha, beta, and gamma frequency bands between the first and second halves of the fatiguing contraction. The data has been averaged over all subjects. Coherence in the beta band was significantly increased during the second half of the contraction compared to that during the first half of contraction.

Comparisons of significant coherence integral in the alpha, beta, and gamma frequency bands during the first and second halves of the fatiguing contraction were presented in Figure 6. The wilcoxon test was used to compare the differences in the significant coherence integrals, and the results revealed that significant coherence integral results in both the alpha (*P* = 0.018) and beta (*P* = 0.005) frequency bands were significantly higher during the second half of the fatiguing contraction compared to those during the first half of the contraction. No significant difference was found between the first and second halves of the fatiguing contraction in the gamma band (*P* = 0.241) among the stroke group and in the alpha (*P* = 0.158), beta (*P* = 0.480), and gamma (*P* = 0.814) bands among the healthy group.

## Discussion

In the present study, we examined the influence of fatigue on the antagonistic muscle coupling of limbs with spasticity induced by stroke. The sEMG activation and coherence between antagonist muscles were compared between the first and second halves of the 30% maximal-level sustained fatiguing isometric elbow flexion contraction between patients with post-stroke spasticity and healthy adults. We found that the intermuscular coherences between the antagonistic BB and TB muscles in the alpha and beta frequency bands were significantly increased during the second half of the contraction compared to those during the first half of the contraction in the stroke group. As far as we know, this is the first study conducted to examine the effect of fatigue on antagonistic muscle coupling of limbs with stroke-induced spasticity.

It may be argued that the reliability of the results may have been reduced by cross-talk contamination, which has been widely studied in relevant studies of antagonist muscle coactivation activities (Lowery et al., 2003; Farina et al., 2004; Wu et al., 2017). In the current research, we adopted an isometric low-force muscle contraction with only a 30% maximal voluntary contraction (MVC), which may be helpful in reducing cross-talk (Farmer et al., 2007). In addition, the size of the electrode adopted in this study was much smaller (with a diameter of 6 mm and area of 28 mm^2^) than the most commonly used electrode (with a diameter of 10 mm and area of 79 mm^2^), which has been suggested to reduce cross-talk (Jaskolska et al., 2006). In addition, EMG rectification has been conducted before coherence analysis according to the recommendation of previous studies, and thus, the influence of cross-talk can be restrained (Farina et al., 2013). EMG–EMG crosstalk, when present, produces high levels of coherence across a wide range of frequencies (1–250 Hz) (Farmer et al., 2007). In the current study, in the stroke group, significant coherence values were found in distinct frequency bands mainly below 60 Hz rather than across a wide range of frequencies. In the healthy group, coherence spectra were mainly discovered below 60 Hz and had very similar values between the first and second halves of the contraction at the same frequency point, which is consistent with the results of previous research (Wang et al., 2015). Therefore, it seems that the results in the current study were not significantly influenced by cross-talk.

In this study, the maximal torque of elbow flexion decreased significantly during the post-fatigue contraction compared to that during the pre-fatigue contraction, while the EMG median frequency of the BB muscle during the second half of the contraction showed a significant reduction compared to that during the first half of the contraction. This demonstrated that muscle fatigue of the agonist muscle BB occurred during the sustained elbow flexion contraction. In previous researches, it has been found that post-stroke spasticity increased force inaccuracy and variability and thus increase the movement instability (Carlyle and Mochizuki, 2018). As a result, the cocontraction level of antagonistic muscles showed a significant increased than healthy subjects, which is consistent with this study. In the stroke group, fatigue seemed to increase the activity of the antagonist muscle rather than that of the agonist muscle in post-stroke spasticity group subjects as the coactivation and EMG power of the TB muscle in the beta frequency band showed significant fatigue-related increases, while the EMG power of the BB muscle showed no significant increase in any frequency bands.

EMG-EMG coherence in the beta band has been suggested to be closely related to the common corticospinal drive from the motor cortex to the muscles (Chen et al., 2018), whereas coherence in the alpha band is influenced by multiple factors such as the stretch-reflex, mechanical resonance, and cortical drives (McAuley and Marsden, 2000). In this study, increased coherence in the beta frequency bands between the antagonistic elbow muscles was observed during the second half of the contraction compared to that during the first half of the contraction in the stroke group, indicating a fatigue-related increase in beta band coherence between the antagonistic elbow muscles during the sustained isometric elbow flexion exercise. The enhanced coherence in the beta bands in the stroke group is considered to reflect increased common neural inputs and stronger coupling between motor cortical neurons and the motor units of co-contracted muscles (Keenan et al., 2012). This result is consistent with the findings of previous research involving healthy young men (Wang et al., 2015). This result may indicate increased coupling between synchronized cortical neurons and the motoneuron pool of the BB and TB.

However, no significant coherence was found between the first and second halves of the fatiguing contraction in healthy adults in the current study, which is inconsistent with the results acquired in sustained isometric fatiguing contraction with 20% MVC force level in previous research (Wang et al., 2015). The higher force level of sustained isometric contraction leads to a higher proportion of peripheral fatigue and lower proportion of central fatigue (Gandevia, 2001; Sogaard et al., 2006). On the other hand, the fatigue of the stroke group may be mainly attributed to central fatitue as the low efficiency of motor control attributed to motor impairment, as well as the low force level for peripheral muscle capacity. Therefore, the results of this study seemed to indicate that fatigue-related changes of antagonistic muscles EMG coupling may have close relationship with the degree of central fatigue.

In this study, we have mainly focused on the fatigue-related EMG coupling properties of antagonistic muscles in the population of patients with post-stroke spasticity. As a limitation of this study, the stroke survivors without spasticity have not been acquired and compared. This makes it hard to explicitly demonstrate the direct correlation between spasticity and muscle coupling. Actually, the results in the current study may be close related to cortically originated muscular discoordination, or motor recruitment, rather than spasticity in passive motion. Therefore, whether spasticity may significant influence EMG coupling of antagonistic muscles in stroke patients still need further research.

EMG synchrony within the Piper frequency band is considered a biomarker of functional corticomotor integrity (Clark et al., 2013; Lodha et al., 2017). A number of prior studies have shown that central nervous system disease and functional deficits are linked to reduced EMG synchrony within the Piper frequency band (Petersen et al., 2013; Willerslev-Olsen et al., 2015). Patients with post-stroke spasticity are expected to have diminished EMG synchrony within the Piper frequency band during motor tasks due to the damage of descending corticomotor pathways (Lodha et al., 2017). Therefore, an enhancement in EMG-EMG coherence may indicate better functional integrity of corticomotor tracts during motor tasks for patients with post-stroke spasticity. In previous research, fatigue has been suggested as a training stimulus to promote exercise performance (Gabriel et al., 2001). Therefore, the increase in antagonistic muscle coupling induced by muscle fatigue may provide suggestions for the design of training programs for patients with post-stroke spasticity.

## Conclusion

A significant increase in intermuscular coherence in the beta frequency band between the antagonistic elbow muscles was observed during the second half of the 30% maximal-level sustained isometric contraction compared to that during the first half of the contraction, indicating that muscle fatigue significantly increases the coupling of antagonistic muscles in patients with post-stroke spasticity, which may be related to the increased common corticospinal drive from the motor cortex to the antagonistic muscles. The increase in antagonistic muscle coupling induced by muscle fatigue may provide suggestions for the design of training programs for patients with post-stroke spasticity.

## Data Availability Statement

The raw data supporting the conclusions of this article will be made available by the authors, without undue reservation.

## Ethics Statement

The studies involving human participants were reviewed and approved by the Ethics Committee of the Tongji University. The patients/participants provided their written informed consent to participate in this study.

## Author Contributions

L-JW, X-MY, and W-XN conceived and designed the study. S-JH and X-MY recruited the subjects and collected the basic characteristics of the subjects. X-MY performed the clinical assessment. L-JW, Q-NS, CW, and HY performed the experiments. L-JW, S-JH, X-MY, and W-XN made a contribution to the data analysis. L-JW wrote this manuscript. All authors contributed to the article and approved the submitted version.

## Conflict of Interest

The authors declare that the research was conducted in the absence of any commercial or financial relationships that could be construed as a potential conflict of interest.
